# The Administration of the Lunacy Acts: Are Further Safeguards Needed?

**Published:** 1915-05-08

**Authors:** 


### THE ADMINISTRATION OF THE LUNACY ACTS.

### Are Further Safeguards Needed?

The public possesses a comfortable feeling that it is a very difficult thing for anyone—not obviously insane—to be certified as a lunatic; feels, in fact, that it is practically an impossibility for a person of sound mind to be incarcerated in an institution through the malevolence of enemies or the well-meaning interference of friends. Nevertheless, a case that has recently occurred may well disquiet us as to our safety in this respect. The circumstances have been revealed in a letter to the *Times* (April 8, 1915) from Mr. Holcombe Ingleby, M.P. for King's Lynn, the main points of his narrative being as follows:—

A lady who had conceived suspicions as to the doings of alleged spies in a certain quarter of London decided to lay her case before the authorities, and, calling at a police office, gave an account of the facts on which her beliefs had been based. As a result of this, she was closely examined as to her name, age, and relatives, and after seeing one or two officials, was asked to wait; but instead of being introduced to a higher officer according to her expectations, she was eventually confronted with a female attendant together with a police constable, and asked to take a journey in a taxi-cab. The amazing point is that the end of her ride was an institution none other than the City of Westminster Workhouse, wherein she soon found herself an inmate of the lunacy observation ward. According to her own account the unfortunate lady was then undressed, washed, and medically examined in accordance with the usual routine, and throughout the next two and a half days had to suffer the companionship of lunatics. On the third day a magistrate appeared who soon satisfied himself as to her sanity and ordered her discharge. Subsequently a high official wrote her a letter in the course of which he admitted that there had been absolutely no necessity for her being subjected to such treatment, and expressing great regret for "the inconvenience and distress of mind caused to you by the action of one of my officers."

### Amend Section 20 of 1890 Act.

It is doubtful if any more lamentable episode in the administration of the Lunacy Acts has occurred for many years, and remarkable though it appears, the police seem to have acted well within the limits of the law in casting this unfortunate lady amongst insane inmates of a Poor-Law institution, where, in full possession of her faculties, she was forced to endure the amenities of Bumble and his myrmidons for some fifty-two hours. Those who doubt the legality of proceedings by which a police officer can haul a visitor off to the workhouse in a taxi-cab as an alleged lunatic, have only to read section 20 of the Lunacy Act, 1890. "If a constable, relieving officer, or overseer is satisfied that it is necessary for the public safety or the welfare of an alleged lunatic that the alleged lunatic should, before any such proceedings can be taken, be placed under care and control, the constable, relieving officer, or overseer may remove the alleged lunatic to the workhouse of the Union where the alleged lunatic is, and the master of the workhouse shall . . . receive and relieve, and detain the alleged lunatic therein, but no person shall be so detained for more than three days, and before the expiration of that time the constable, relieving officer, or overseer shall take such proceedings with regard to the alleged lunatic as are required by this Act." The law does not seem to question the ability of any constable, relieving officer, or overseer to judge of an individual's state of mind without reference to medical opinion; but whilst this is so, it can scarcely be doubted that the Lunacy Act requires careful revision. Every physician of experience knows that it is by no means always an easy matter to judge accurately of the state of mind of an excitable person at the first interview when for any reason great emotional feeling has been called up. This being so under such circumstances as have been narrated by Mr. Holcombe Ingleby, a lay official takes upon himself very grave responsibility in attempting to decide as to the sanity of a victim without the support of medical authority. As to section 20 of the Lunacy Act, doubtless it was framed to enable a parish official speedily to bring within control an irresponsible person in an obvious state of mental derangement. Clearly an "alleged lunatic" may be in any state between incoherent raving and mild excitement; be murderous in intent or merely believe that the moon is made of green cheese. In one instance it is highly essential for public safety that machinery should exist for bringing a dangerous lunatic within control, and in the other, whilst the Act may still apply, there need not necessarily be any urgency for putting its provisions in motion.

### How to make Lunatics.

Whilst the law permits of the detention of an alleged lunatic who has been removed to the workhouse infirmary, at the direction of a constable, relieving officer, or overseer, for a period of three days, common sense would suggest that a magistrate should be brought into the matter long before the end of that time. Is it fair to make the alleged lunatic, in an obviously mild case, confront either doctor or magistrate whilst in workhouse clothes, and in the case of women, probably with hair dressed very differently from their usual custom? There are many sane persons who, if kept in a lunacy observation ward for two days, dressed in workhouse garb, and sent to prove their sanity to a stranger, would very likely succeed in giving an entirely wrong impression of themselves. Obviously the more excited they appeared—and who would not be grievously excited under such circumstances?—the worse impression the patient would give. The effrontery of the wrong doers in the case related by Mr. Holcombe Ingleby is to be found in a bill presented to the unfortunate lady for three days' maintenance and the doctor's fee.

